# Supplementary material for: Human DNA polymerase ε is a source of C>T mutations at CpG dinucleotides
Source: Nat Genet. 2024 Oct 10;56(11):2506–16. doi: 10.1038/s41588-024-01945-x (PMC11549043; doi:10.1038/s41588-024-01945-x)
Supplement: Supplementary file 1 — Supplementary Notes 1–13. [file 41588_2024_1945_MOESM1_ESM.pdf]

nature genetics

Supplementary information https://doi.org/10.1038/s41588-024-01945-x

Human DNA polymerase ε is a source of C>T mutations at CpG dinucleotides

In the format provided by the authors and unedited

Supplementary Notes for

Human DNA polymerase ε is a source of C>T mutations at CpG dinucleotides

Table of Contents

Supplementary Tables 1
Supplementary Notes 3
Supplementary Note 1: Barcodes, Alignment and Coverage 3
Supplementary Note 2: Background Errors 3
Linear amplification and dual barcoding 3
Minimised heating 7
Background subtraction 7
Supplementary Note 3: PER-seq optimisation 9
Supplementary Note 4: Deamination in ssDNA 9
Supplementary Note 5: Quality control of Pol ε activity and filling 12
Supplementary Note 6: Order of MMR loss and POLEd variants 13
Supplementary Note 7: Multi-mutations 14
Supplementary Note 8: Pol ε errors in TP53 hotspots 15
Supplementary Note 9: Discussion of mechanisms of P286R mutagenesis 17
Supplementary Note 10: Mutational signature SBS5 18
Supplementary Note 11: PER-seq, plasmid preparation 19
Supplementary Note 12: Introducing P286R mutation in mESCs 20
Supplementary Note 13: PER-EXTRACT-seq 21
References used in the supplementary materials 21

Supplementary Tables

Supplementary Table 1: Forward and reverse adapters and the ROI sequences.

Supplementary Table 2: Positions of artificially introduced mutations.

Supplementary Table 3: PER-seq samples.

Supplementary Table 4: PER-seq measured error signatures of KLENOW-EXO$^-$, KAPA-U+, POLE-P286R, POLE-EXO$^-$, and POLE-WT.

Supplementary Table 5: Human cancer samples.

Supplementary Table 6: Pathogenic mutations in the exonuclease domain of POLE and POLD1, used in the definition of POLEd and PROF samples (see Methods).

Supplementary Table 7: BS-seq and H3K36me3 datasets.

Supplementary Table 8: Oligonucleotides for cloning of guide RNA.

Supplementary Table 9: Single-stranded oligonucleotide (ssODNs) templates for homology-directed repair.

Supplementary Table 10: Primers used to amplify and sequence mutations resulting in P286R in POLE.

Supplementary Table 11: PER-EXTRACT-seq samples.

Supplementary Notes

Supplementary Note 1: Barcodes, Alignment and Coverage

The sample barcodes ("pads") were 6bp long in total on average, with one part being on read1 and the other part on read2. The sample barcodes were designed using genetic algorithms to maximise library complexity and distance between samples.

Needleman-Wunsch algorithm was used to align the reads. The penalty parameters were -1 for a mismatch of a base with low-quality base call (PHRED score < 21), -2 for a mismatch of a high-quality base call (PHRED score ≥ 21), and -2 for an indel. In cases of a read-pair overlap, the read1 values were used, unless for bases in the overlap with low quality in read1 and high quality in read2. Since single base substitutions (SBS) were the major focus in this study, reads with indels were filtered out, to ensure that the SBS calls are not confounded by neighbouring indels, wrong alignment of nearby indels, or other indel-related issues. For variant calling, only bases with a PHRED score ≥ 21 (in at least three independent linear copies) were considered. Only molecules with at least three linear copies (each with a different unique linear-copy identifier) were used. Each sample had 1.4 million well-covered molecules in median (Extended Data Table 1).

Supplementary Note 2: Background Errors

During PER-seq, assay-specific background errors can result from the following sources:

1. Errors can be produced during the linear and exponential amplification.
2. Errors can be produced during Illumina sequencing.
3. DNA damage can happen during the entire assay. E.g., the single-stranded gap in plasmids can accumulate damage, including spontaneous deamination of cytosine or 5-methylcytosine. Similarly, damage can happen during the short periods of heating during the library preparation.
4. The parental plasmid can carry some background mutations introduced in *E. coli* that escaped the repair.

PER-seq is designed to minimise/account for all these four sources of errors, and subtract any potential remaining errors, in the following steps: (a) linear amplification and dual barcoding, (b) minimised heating, (c) background subtraction. Each step is described in detail below.

Linear amplification and dual barcoding

All variants need to be present in three independent linear copies – with the same unique molecular identified but with different unique linear-copy identifiers (note that this differs from the major use

in the original MDS protocol[1], as the dual-barcoding showed to be an important step for PER-seq during our optimisation process). As explained below, the probability of a variant resulting from a sequencing or amplification error is lower than 1e-9.

The table below describes the parameters of the PER-seq protocol relevant for estimating the false-positive rate (see Supplementary Note 3 for explanation of the PER-seq optimisation of some of the parameters):

		Upper or lower bound	
L	Number of linear copies (sufficiently covered and with sufficient base quality) for a given molecule	≤ 10	We used 10 linear rounds for the first library, and further decreased this to 7 for all subsequent libraries
E_A	Error rate of the amplification DNA polymerase	≤ 1e-5	An upper bound; in reality, this is expected to be much lower (e.g., < 2e-6 for Kapa-U+)
E_S	Error rate of Illumina sequencing	≤ 1e-3	Upper bound estimate[2]
X	Number of exponential rounds	≤ 19	Values range between 9 and 19 rounds
V	Required minimal variant allele frequency	≥ 70%	
M_L	Minimum number of linear copies required	≥ 3	
M_R	Minimum number of reads per linear copy required	≥ 1	
PA_{read}	Probability of an amplification error in a given read and position	≤ 2e-4	Explained below
PA_{linear}	Probability of an amplification error in a given linear copy and position	≤ 2e-4	Explained below
$PA_{molecule}$	Probability of an amplification error called as a variant in a given molecule and position	≤ 1e-10	Explained below

$PS_{molecule}$	Probability of a sequencing error called as a variant in a given molecule and position	≤ 1e-9	Explained below

Amplification errors in a given **read** could have happened during the one linear round, or one of the *X* exponential rounds. The probability for each of the reads to have an amplification error at a given position can be therefore computed as the complement to no amplification errors happening during the 1 linear and *X* exponential rounds:

$$PA_{read} = 1 - (1 - E_A)^{(X+1)} \leq 1 - (1 - E_A)^{20} \leq 1.9998e - 04$$

For a variant called in one **linear copy** to result from an amplification error(s), at least 70% (*V*) of the reads within that linear copy would need to carry that amplification error. Since the minimum number of reads per linear copy required is $M_R = 1$, this represents also the upper bound for PA_{read}. When more than 1 reads are present, the probability of an amplification error is either the same (as the error could have happened in the linear amplification or the shared exponential rounds of these reads) or lower (if the amplification error happened in two independent branches of the exponential amplification). Therefore:

$$PA_{linear} \leq PA_{read} \leq 1.9998e - 04$$

For a variant called in a given **molecule** to result from amplification errors, the same error would need to occur at the same position in at least three (M_L) independent linear copies (or during their exponential amplification). More specifically, the error would need to occur in at least 70% (*V*) of the *L* linear copies, which corresponds to at least *k* successes of *L* attempts, where *k* spans from *ceil(V*L)* to *L*. For example, when *L* is 4 linear rounds, then *k* has values 3 (as ¾=0.75 ≥ 0.7) and 4. This can be computed as follows:

$$PA_{molecule} = \sum_{k=(ceil(V*L))}^{L} \binom{L}{k} (PA_{linear})^k (1 - PA_{linear})^{n-k}$$

The values of $PA_{molecule}$ are below 1e-10 for all scenarios with at least L= 3 linear copies (Supplementary Figure 1).

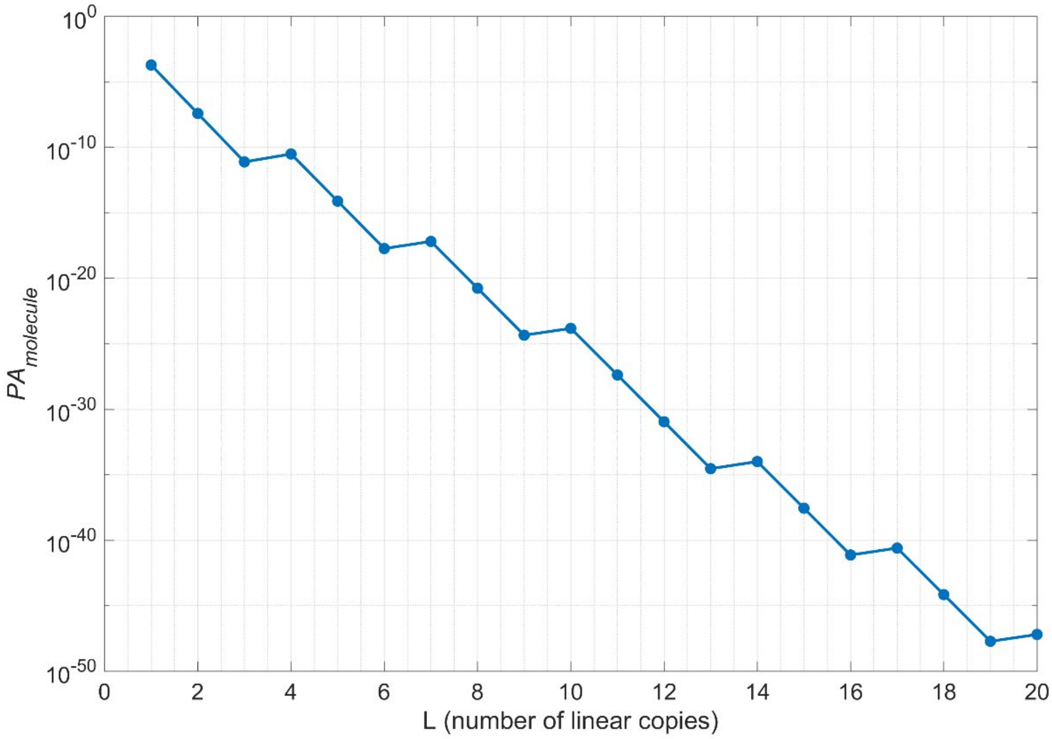

Supplementary Figure 1: Probability of a variant called in a given molecule to result from amplification errors (PAmolecule) shown for different values of linear amplification copies recovered in the molecule (L). The y-axis is shown in a log_{10}-scale.

The upper bound for the probability of a called variant coming from Illumina sequencing error(s) is:

$$PS_{molecule} = (E_S)^L \leq (1e\text{-}3)^3 = 1e\text{-}9$$

Minimised heating

The entire protocol has been optimised to reduce heating (and thus heating-induced damage), especially at high temperatures. Any damage that happens late enough (e.g., in the exponential amplification) has low probability to be called as a variant for the same reasons as explained above for the amplification errors. However, damage that happens early (e.g., in the single-stranded DNA of the gapped plasmid, or during the "linear 0" step of the amplification part) can contribute to assay-specific background of PER-seq, and is subtracted in the "background subtraction step" (see below).

Background subtraction

In order to subtract any potential remaining background errors, we use a background subtraction approach. In particular, we sequence both strands of the parental plasmids ("parental template strand" and "parental daughter strand") that have never been gapped/filled by Pol ε and both the "template strand" and "daughter strand" of the filled plasmids (Fig. 1a, Extended Data Fig. 1).

Variant calling after background subtraction is then defined as:

Daughter – Gapping bg. – PD bg.

where:

- Daughter = variants called in the "daughter strand"
- Gapping bg. = variants called in the "template strand" minus "parental template strand" (these include potential damage that happened to the template strand while gapped)
- PD bg. = variants called in the "parental daughter strand" (including mutations introduced in *E. coli*, as well as any potential damage that happened during library preparation)

For methylated samples, the correctly matched samples need to be subtracted:

Daughter^{M} – Gapping bg.^{M} – PD bg.^{U}

where:

- Daughter^{M} = daughter strand of the *methylated* plasmid
- Gapping bg.^{M} = variants called in the *methylated* "template strand" minus *methylated* "parental template strand" (these include potential damage that happened to the template strand while gapped)
- PD bg.^{U} = variants called in the *unmethylated* (mock-treated) "parental daughter strand", as the true daughter strand is also unmethylated at the amplification stage of PER-seq

The background subtraction process is then performed on the level of the strand-specific 192-channel error spectrum (number of errors in the given trinucleotide, divided by the number of such trinucleotides in the ROI). The reverse complement error spectrum of the template and parental template strands is used, to correctly account for the error direction.

The measured median gapping and parental background values were 4.2e-6 and 3.7e-6, respectively (Supplementary Figure 2). The overall values did not significantly differ between the methylated and mock-treated samples.

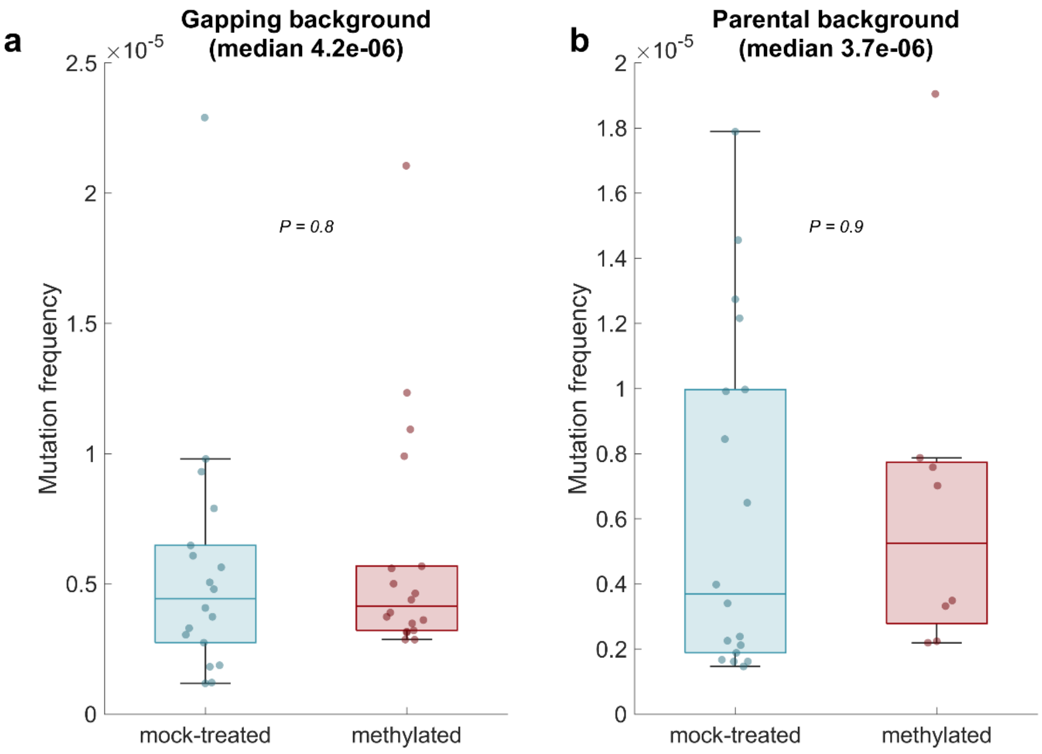

Supplementary Figure 2: Distribution of the measured average gapping background (a) and parental background (b) in mock-treated (teal) and methylated (dark red) samples. N: 18 (a, mock-treated), 17 (a, methylated), 18 (b, mock-treated), 8 (b, methylated). Two-sided two-sample t-test with uneven variance P-values are shown on top of the boxplots to compare the values in methylated and mock-treated samples. Boxplots are plotted with the MATLAB function boxchart (see Methods).

Importantly, the background subtraction can account for potential spontaneous deamination that happened during gapping or library preparation, to ensure that the observed CpG>TpG mutations are true Pol ε errors and not products of spontaneous deamination (Extended Data Fig. 7).

Supplementary Note 3: PER-seq optimisation

Some of the PER-seq protocol parameters were changed after the first novaseq library in order to increase coverage yield (well-covered molecules), further reduce heating-associated damage, and ensure that unused reverse adapter do not get used during the exponential amplification (which would lead to a single linear copy being represented by reads with multiple different reverse barcodes). The fully optimised protocol V2 is described in the methods section, with the difference summary being listed in the table below:

Parameter/step	V1 (validation1, novaseq1)	V2 (novaseq3, novaseq4, novaseq5)	Reason
Amplification polymerase	KapaU+	Q5U	To increase coverage yield
Each sample pooled from two preparations	No	Yes	To increase coverage yield
Number of linear rounds	10	7	To reduce heating-associated damage
PCR conditions optimised (shortened)	No	Yes	To reduce heating-associated damage
Number of exponential rounds	Fixed (19 rounds)	Minimal necessary (see Methods)	To reduce heating-associated damage
Removal of unused rv adapters	No	Yes	To ensure that rv adapters do not get re-used

Supplementary Note 4: Deamination in ssDNA

Any ssDNA will accumulate deamination events. We designed the PER-seq protocol with this in mind and any deamination pattern that was introduced during the gapping, filling, or library preparation will be detected therein and subsequently subtracted.

We always sequence the “parental” sample, that is the plasmid *before* the gapping & filling. We detect some C>T (T:G) mismatches in the parental samples, and their frequency matches the values we would expect based on the estimated duration the DNA spends as ssDNA during the library preparation and the known rates of C and 5mC deamination in ssDNA[3] (Supplementary Figure 3a-b; compare the measured data in boxplots and expected values in lines). We optimised the protocol to minimise heating-associated cytosine deamination during library preparation, which resulted in a substantial

reduction of C>T deamination events emerging from construction of sequencing libraries (compare Supplementary Figure 3a and Supplementary Figure 3b).

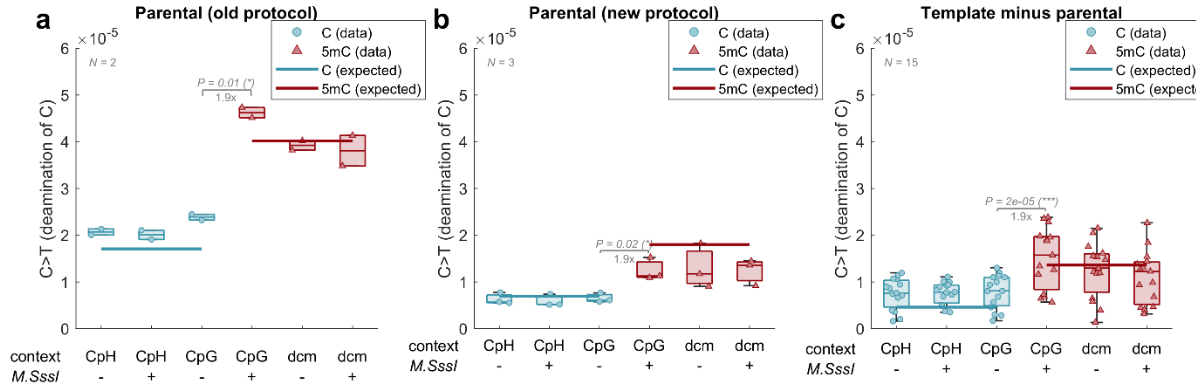

Supplementary Figure 3: Background C>T errors in parental samples from the old protocol (validation1 and novaseq1 libraries), new protocol (novaseq 3-5), and on the gapped template. N = 2 (a), 3 (b), and 15 (c). The lines represent expected numbers based on the deamination rate of ssDNA[3] and durations that ssDNA spends at different temperatures. A paired two-sided t-test was used to compare the values between the groups and the ratio of the medians is shown below the significant P-values. Boxplots are plotted with the MATLAB function boxchart (see Methods).

We also always specifically sequence the template strand of the ROI *after* filling by the respective polymerases. This is what we refer to as the "template". The difference between the template and the parental sample will capture deamination events that happened while the template was ssDNA between gapping and filling. Indeed, the C>T (T:G) mismatches we detect correspond to the duration the substrate spends as ssDNA before filling (Supplementary Figure 3c).

This demonstrates that we can accurately detect deamination damage that happened both before and during library preparation. This is what contributes to the "background errors" of the assay. Crucially, we subtract these background errors from the values observed on the daughter strand, do ensure that they do not contribute to the POLE error spectra.

It is also worth pointing out that these background errors (e.g., cytosine deamination in gapped template) remain similar for POLE-WT, POLE-EXO$^-$, and POLE-P286R (Supplementary Figure 4).

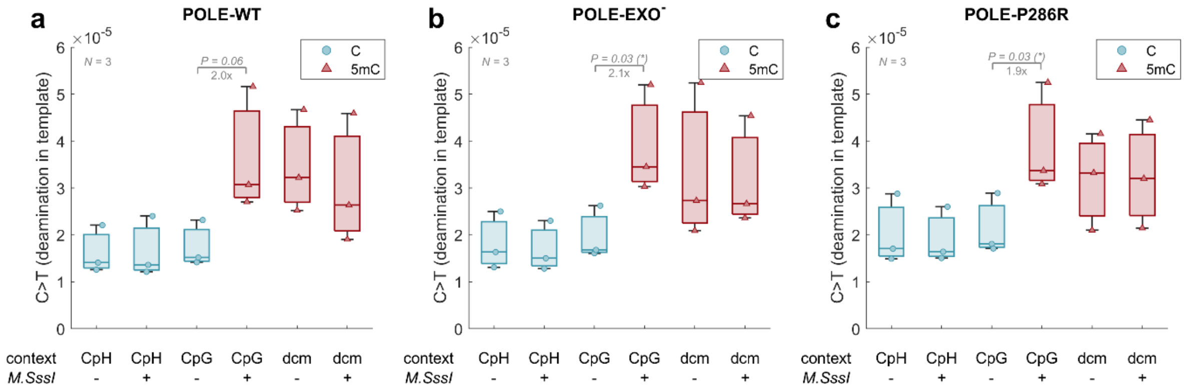

Supplementary Figure 4: Background C>T mutations in the template samples (corresponding to cytosine deamination during gapping, filling, and library preparation). For clarity, the raw mutation frequencies (without background subtraction) in the TP53 ROI are shown here. A paired two-sided t-test was used to compare the values between the groups and the ratio of the medians is shown below the significant P-values. Boxplots are plotted with the MATLAB function boxchart (see Methods).

This is in stark contrast to the true C:dA misincorporations detected in the daughter strand (i.e., misincorporation of dA opposite C by Pol ε), which show a ca. 6-fold increase in POLE-EXO^{-} and over 12-fold increase in POLE-P286R, compared to POLE-WT (Supplementary Figure 5).

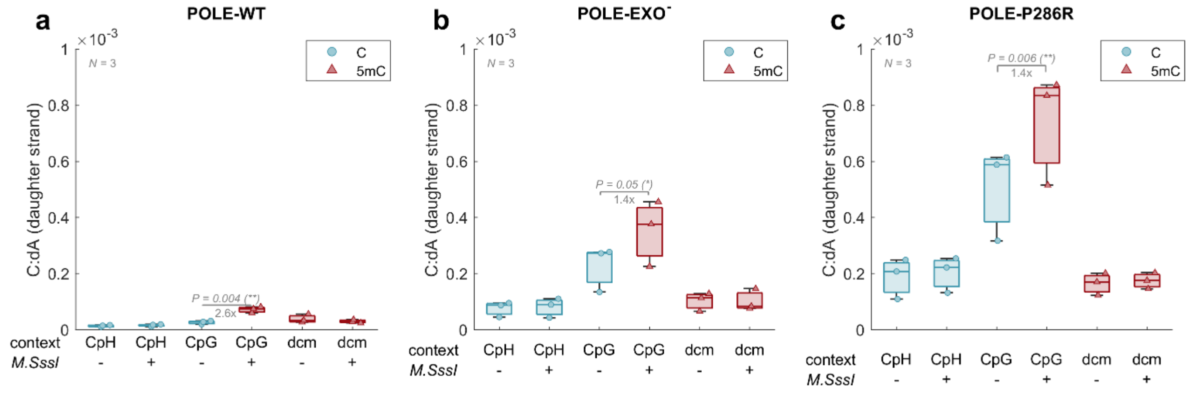

Supplementary Figure 5: Mutations in the daughter samples (C:dA misincorporation). For clarity, the raw mutation frequencies (without background subtraction) in the TP53 ROI are shown here. A paired two-sided t-test was used to compare the values between the groups and the ratio of the medians is shown below the significant P-values. Boxplots are plotted with the MATLAB function boxchart (see Methods).

This shows clearly that the deamination rate of the template strand cannot explain the observed high CpG>TpG (C:dA) rate in the daughter strands and that these represent true mis-incorporations of adenine opposite template 5mC by the polymerase.

Finally, we would like to clarify that both the daughter and the template strands get sequenced only from those molecules that were completely filled, ensuring that enzyme prep activity does not

confound this. Also as discussed above, we have measured specific activity of different enzyme batches to ensure that our enzyme purification is of consistent quality.

Supplementary Note 5: Quality control of Pol ε activity and filling

The tables below represent percentage of unfilled plasmids:

Filling 1	Wt	exo-	P286R
TP53 unmethylated	ND	<10%*	ND
TP53 methylated	11.1%	<10%*	ND
DNMT1 unmethylated	0.9%	7.8%	ND
DNMT1 methylated	9.8%	12.2%	ND

Filling 2	wt	exo-	P286R
TP53 unmethylated	6%	12.6%	8.6%
TP53 methylated	<10%*	2%	<10%*

Filling 3	wt	exo-	P286R
TP53 unmethylated	ND	4.6%	ND
TP53 methylated	0.2%	3.1%	ND

Notes:

- The numbers represent the percentage of the template remaining unextended, calculated by scanning the gels and then using Image J on the raw TIFFs.
- ND = none detected (above background).
- * Exact data not available.

The table below shows specific activity measurements for each enzyme prep (normalised to wt):

	wt	exo-	P286R
First prep	1	1.01	0.88
Second prep	1	1.57	1.49

Notes:

- Specific activity was measured by performing extension reactions as described previously[4]. Briefly, 20 fM of DNA polymerase was combined with excess of A2 substrate

(CGCTGGCCGTAGTCTTCCAACGTCGTGACTGGGAAAA) annealed to C700/800 primer (TTTTCCCAGTCACGACGTTG) and incubated over a time course (1-8 min). Extension products were resolved using denaturing electrophoresis and quantified using Licor Odyssey CLx imaging system. Values were normalised to activity of *wt* protein.

The table below shows mapping between enzyme prep, filling number, and NovaSeq libraries:

library	ROI	Enzyme prep	Filling
novaseq1	TP53	1	1
novaseq1	DNMT1	1	1
novaseq3	TP53	2	2
novaseq4	TP53	2	3

Supplementary Note 6: Order of MMR loss and POLEd variants

We have shown that the PER-POLE-P286R error signature closely resembles mutational profile of patients with combined POLEd&MMRd. However, it is known that the order of MMR loss and acquisition of the POLEd variant results in slightly different mutational profiles[5,6]. Comparing our PER-seq measurements with profiles of tumours with known order of MMR loss and POLEd variant[6], we show that PER-POLE-P286R best corresponds to profiles of cancer samples where MMR loss precedes POLEd variant (Supplementary Figure 6).

Indeed, seven of the 17 POLEd & MMRd samples included in our study have a germline biallelic MMR deficiency (bMMRd), and thus the MMR loss preceded acquisition of the POLEd variant in them. Additional six samples have a stop-gained mutation in one of the MMR genes (*MSH6*, *PMS2*, *MSH2*, or *MLH1*) with VAF higher than (or in one case similar to) the VAF of the POLE variant, in line with the MMRd preceding POLEd. Of the remaining four samples, two had high (>50%) and one fairly high (27%) *MLH1* promoter methylation, and one did not have methylation values available. In these fours samples, it is hard to determine the order of the MMRd loss, however, the rest of the cohort support the conclusion that the PER-POLE-P286R error signature best resembles mutational profile of MMRd loss preceding POLEd mutation.

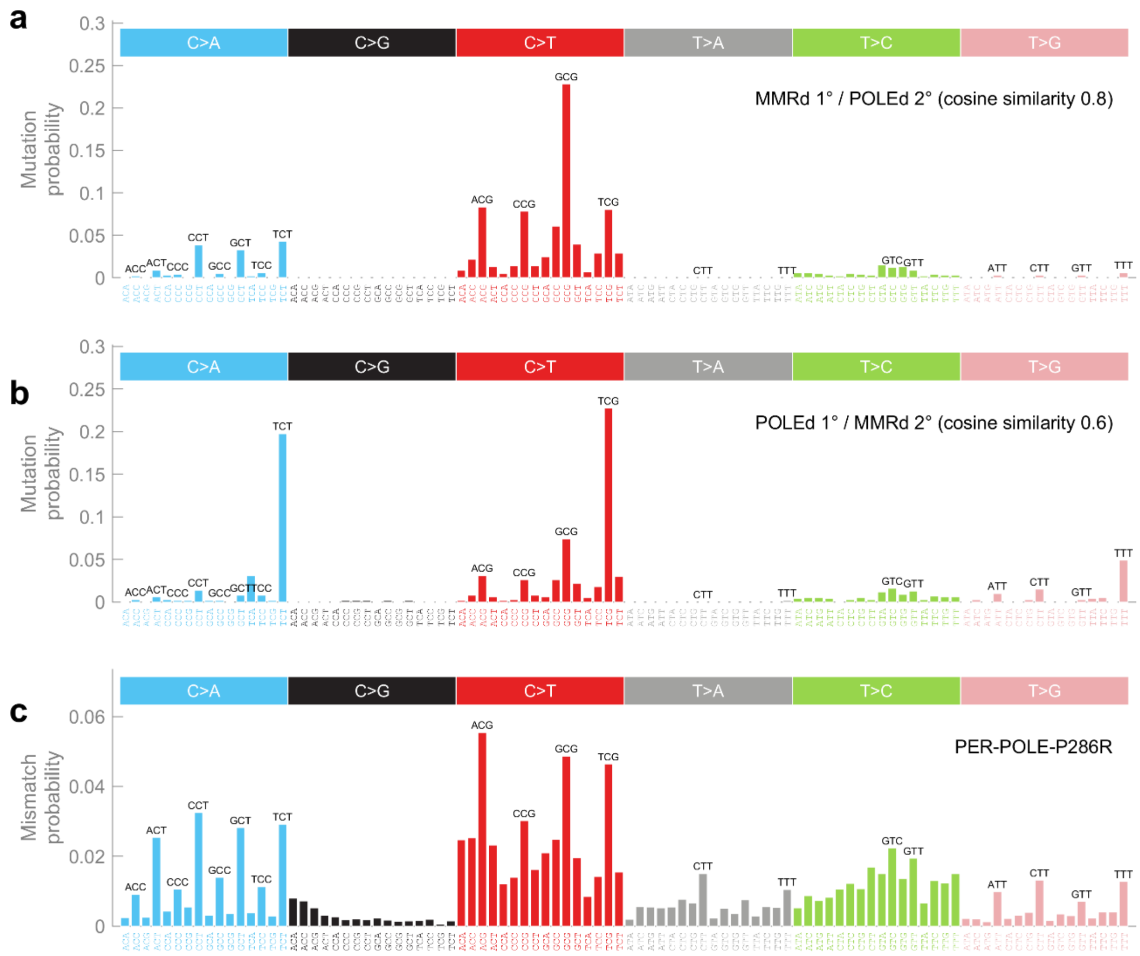

Supplementary Figure 6: Comparison of the PER-POLE-P286R error signature with mutational profiles of POLEd&MMRd samples by the order of MMR loss and POLEd variant acquisition. a, Profile of samples where MMR loss occurred first. b, Profile of samples where POLEd variant occurred first.c, Error signature of PER-POLE-P286R. Values for a and b are based on the Fig. 5A of the study by Campbell et al.[6]

Supplementary Note 7: Multi-mutations

Here, we analysed how frequently does Pol ε introduce more than one CpG>TpG (C:dA) errors in the same molecule (single substrate filling). We observed that the PER-POLE-P286R and PER-POLE-EXO$^-$ make multiple mistakes in the same molecule with a slightly higher frequency than expected by chance (Supplementary Figure 7). The expected values were computed using permutation testing of the observed CpG>TpG mutations in the given sample. For example, in PER-POLE-P286R, the median expected fraction of molecules with multiple CpG>TpG mutations is 1.2e-5, while the observed frequency is 1.7-fold higher. In the entire dataset, we detected only two molecules with *three or more* CpG>TpG mutations, as expected given the extremely low probability of such a scenario. Altogether, our results suggest that when PER-POLE-EXO$^-$ and PER-POLE-P286R make an error, there is an increased chance of another error happening in the same molecule. However, the frequency of

multiple CpG>TpG mutations in the same molecule are still very rare (only 0-50 molecules out of millions of molecules).

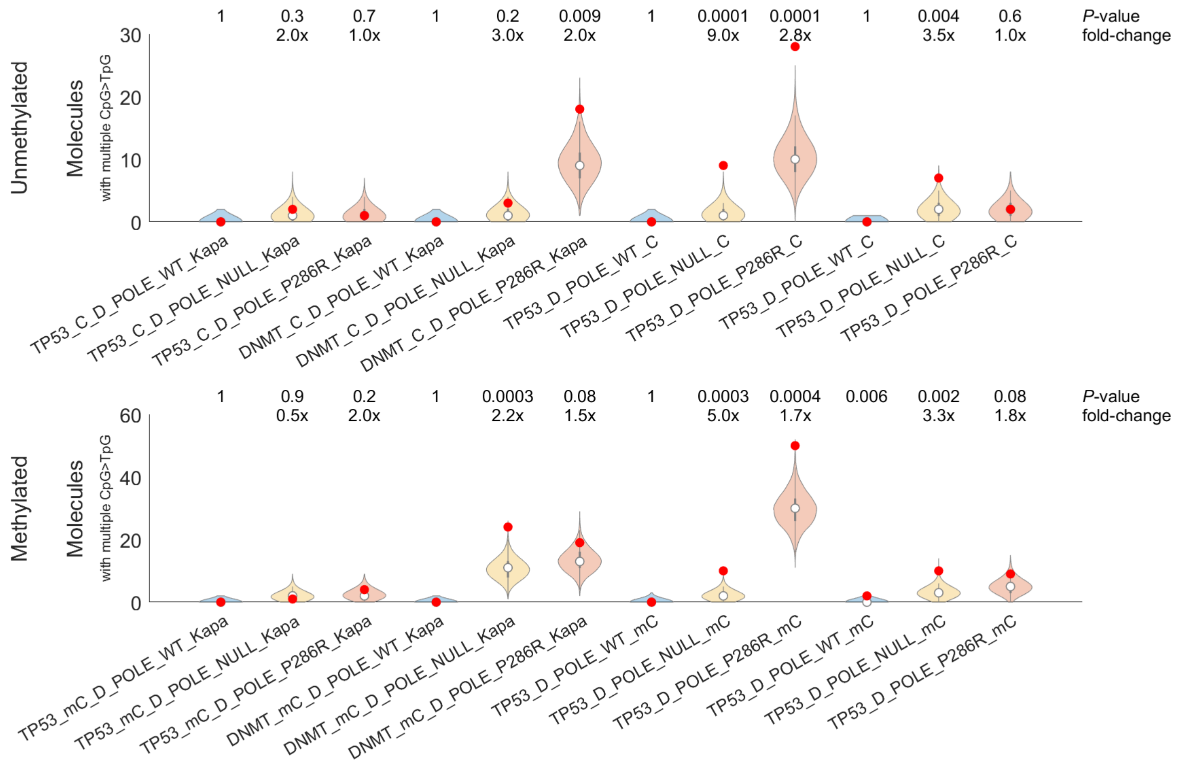

Supplementary Figure 7: Number of molecules with multiple CpG>TpG polymerase errors detected by PER-seq in unmethylated (top) and methylated (bottom) samples. The violin plots represent simulated data (N = 10,000 iterations), the red dots represent real measurements for individual samples. The two-sided permutation test p-value and fold-change of observed vs. median expected values are shown on top.

Supplementary Note 8: Pol ε errors in TP53 hotspots

The TP53 ROI covers the entire exon 8 of the canonical transcript ENST00000269305. This exon comprises three of the top 5 TP53 deleterious mutation hotspots, including the most mutated amino acid (R273). To establish whether Pol ε errors might contribute to the generation of TP53 hotspot mutations, we identified deleterious TP53 mutations within the ROI region from TCGA data (https://portal.gdc.cancer.gov/analysis_page?app=MutationFrequencyApp, deleterious defined as high impact VEP or probably/possibly damaging PolyPhen prediction). For each position in the ROI, we selected the (predicted) deleterious variant with the highest allele frequency (if one exists).

Strikingly, the three best-known TP53 mutation hotspots in our ROI show extremely high Pol ε error rates in our PER-seq experiment (Supplementary Figure 8a). The R273H mutation, due to a C>T mutation in a CpG context, showed the highest PER-seq error rate, due to a mC:dA misincorporation. Notably, the PER-seq derived error frequency at loci that cause deleterious TP53 mutations

significantly correlates with the frequency with which the corresponding mutation is seen amongst TCGA patients (Spearman correlation r -0.6, p 1e-6, Fig. Supplementary Figure 8b). Finally, we searched for TP53 mutations (covered in this ROI) in POLEd samples (including POLEd&MMRd samples). We found four TP53 hotspots mutated in the POLEd samples (R273H, R273C, R282W, and R267W), and all of them had very high frequency in our PER-seq measurements (Supplementary Figure 8a, blank squares). In summary, our data suggest that Pol ε errors contribute to the generation of some of the most important cancer driver hotspots in the TP53 gene.

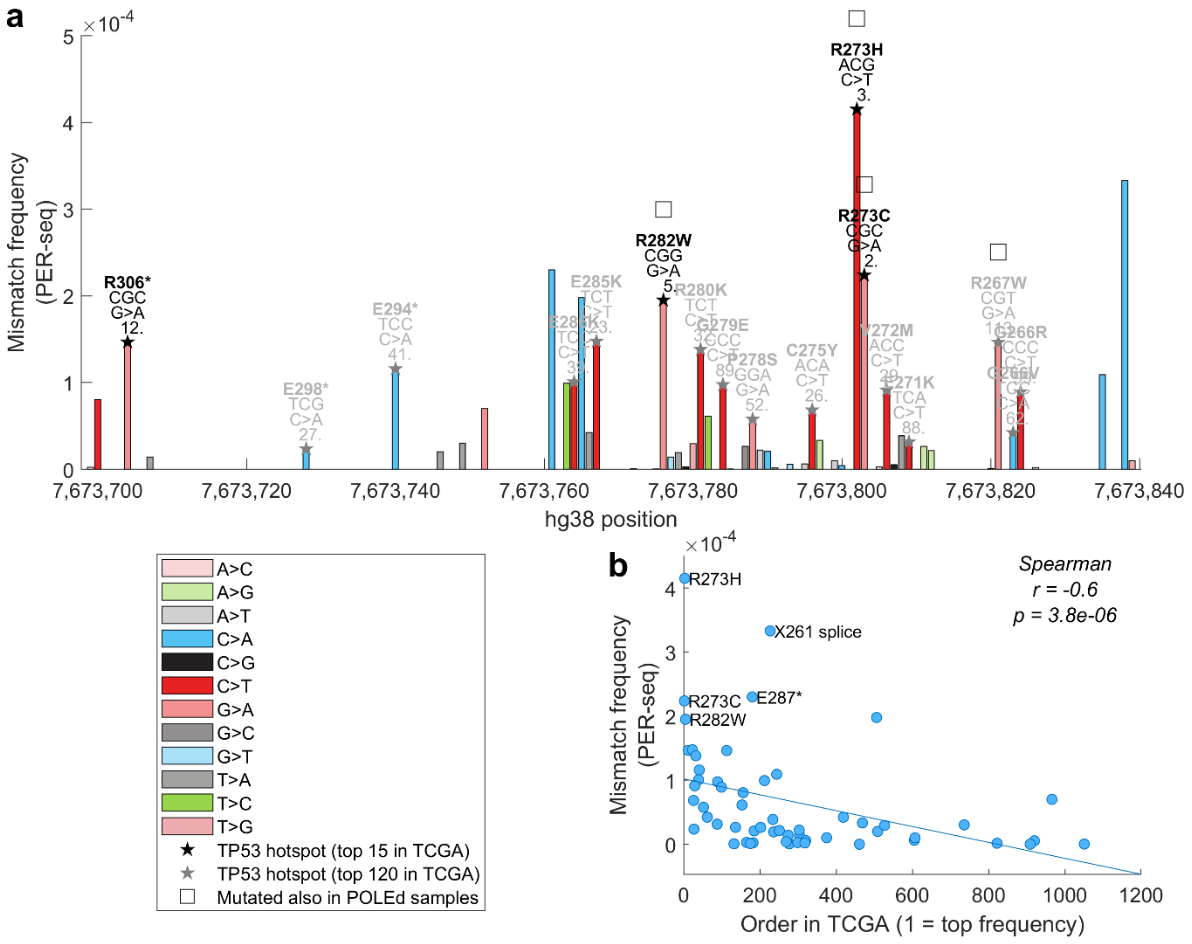

Supplementary Figure 8: Comparison of Pol ε errors in the TP53 ROI measured by PER-seq and mutation hotspots in TCGA. a, The average Pol ε errors measured by PER-seq on methylated template DNA of the TP53 ROI. Only deleterious variants are shown here. The type of base change is colour-coded. The top 15 deleterious TP53 hotspots (across the entire gene) are denoted by black star, and the top 120 hotspots by grey star. Variants detected in six POLEd&MMRd samples in this study are denoted by empty square. The annotations above the top hotspot bars represent: the amino acid change, sequence context, base-change, and deleterious TP53 hotspot rank. b, The PER-seq error frequency plotted against the order (rank) of deleterious TP53 hotspots in TCGA (lowest rank represents highest frequency in TCGA). Two-sided Spearman correlation coefficient and p-value are shown in the top right corner.

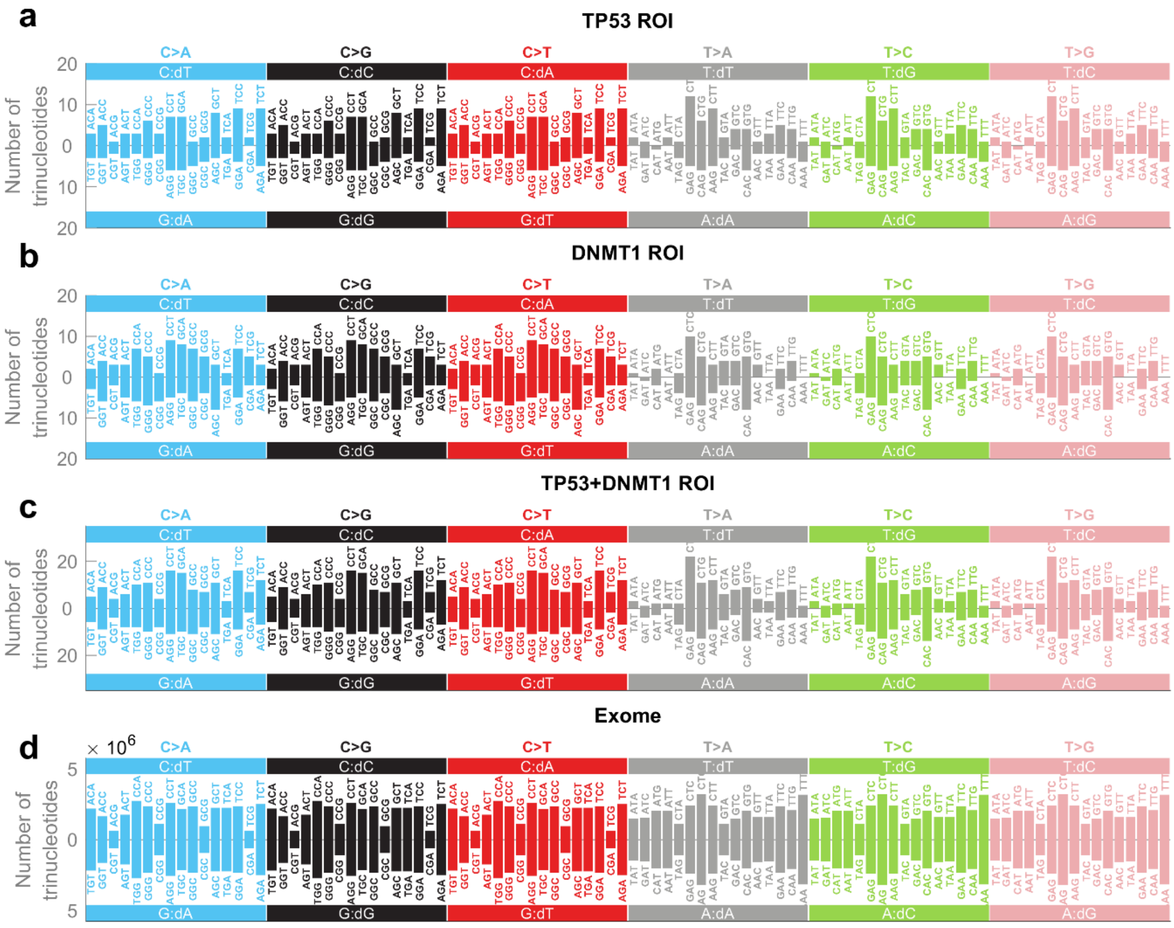

Supplementary Figure 10: Occurrence of trinucleotides in the TP53 ROI (a), DNMT1 ROI (b), both ROIs together (c), compared to the entire human exome.

Supplementary Note 9: Discussion of mechanisms of P286R mutagenesis

The most common pathogenic somatic mutation in Pol ε results in proline 286 substitution with arginine (P286R). In fact, POLE-P286R is the most frequent variant in general in colorectal and endometrial cancers[7–9], with frequency as high as ca. 7% of early-onset colorectal cancers[10]. The exact nature of how the defective enzyme contributes to the high mutational load is an area of active investigation[11–15]. For example, it was proposed that the arginine interferes with DNA entry into exonuclease side in a distinct mechanism of action from substitutions inactivating catalytic ability of exonuclease domain[13].

We show that Pol ε P286R not only generates 27 times more mutations than *wt* Pol ε, but also 2.1 times more mutations than an exonuclease deficient enzyme. This demonstrates that P286R is not simply reducing the proofreading ability of the exonuclease domain. *In vivo*, the equivalent mutation to human P286R, when compared to a loss of Pol ε exonuclease activity, was also found to result in a much stronger mutator phenotype in fission yeast[11,14] and shorter survival in mice[16,17]. In contrast, the

equivalent P301R mutation in *Saccharomyces cerevisiae* Pol ε was reported to produce a lower error rate than the exo-null mutant enzyme *in vitro*[12], probably because the yeast P301R mutant enzyme retains more exonuclease activity compared to its human counterpart, however there might be other species-specific differences as well[4,8,12]. While we cannot yet rule out that other factors, such as disruption of orchestrated repair, may be contributing to the hypermutation phenotype of Pol ε P286R, our data are compatible with the previously postulated hypothesis that P286R represents a gain-of-function mutation.

Supplementary Note 10: Mutational signature SBS5

The decomposition of the PER-POLE-P286R error signature into COSMIC SBS signatures showed a substantial contribution by SBS5 (Fig. 2d). The cause of SBS5 is currently unexplained. Our data raise the possibility that polymerase errors are involved in the aetiology of SBS5. This would agree with the clock-like properties of SBS5[18,19]. We have explored this possibility and compared the SBS5 exposure in different cancer samples using SBS exposures reported in the PWCAG study[19], downloaded from the PCAWG ICGC data portal. Interestingly, the highest burden of SBS5 can be indeed observed in POLEd and in MMRd cancer patients, showing more than 17-fold increase over exposures in patients proficient in POLE and MMR (Supplementary Figure 9). This supports a possibility that polymerase errors contribute to SBS5.

However, SBS5 has a very "flat" profile, and is therefore difficult to distinguish from other signatures with a relatively uniform mutation rate across sequence contexts[70]. It is therefore possible that the "flat" component in PER-POLE-P286R is independent of SBS5. Finally, two of the distinguishing T>C peaks in SBS5 overlap with the two trinucleotides (TAT and AAT) not covered in our currently used two ROIs (Supplementary Figure 10). Therefore, future research will be needed to determine whether polymerase errors might contribute to SBS5.

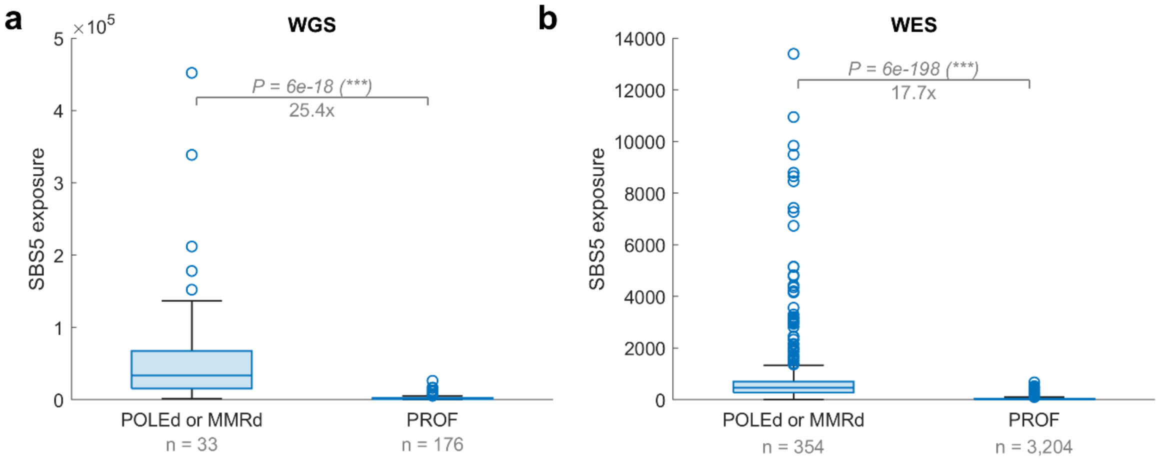

Supplementary Figure 9: Exposure to SBS5 in POLEd or MMRd cancer patients in comparison with PROF (POLEp & MMRp) patients. The SBS5 exposures have been downloaded from the PCAWG ICGC data portal and matched to the samples used in this study. For a fair comparison, only cancer types with POLEd or MMRd samples are shown here in both groups. A two-sided Mann–Whitney U test (rank-sum test) was used to compare the groups. Boxplots are plotted with the MATLAB function boxchart (see Methods).

Supplementary Note 11: PER-seq, plasmid preparation

Two regions of interest (ROIs) were used for PER-seq. The length of the ROI is up to 300bp in total (including priming regions and barcode), so that it is fully covered by 150bp paired-end sequencing. We chose natural sequences that exist in the human genome to avoid potential artefacts which could emerge in an artificially designed sequence. Both ROIs cover an exon. The two ROIs cover a wide spectrum of trinucleotides, with most being represented multiple times (Supplementary Figure 10). The first ROI was chosen from *TP53* gene (hg19 chr17:7,576,947-7,577,207), because it contains position frequently mutated (CpG>TpG) in cancer. The second ROI comprised 260bp of the CpG island of the *DNMT1* gene (hg19 chr19:10,252,661-10,252,920) and it contains 16 CpGs. Each ROI was amplified from human genomic DNA using primers containing *HindIII* and *SacI* restriction sites before cloning PCR products into a pUC19 vector modified by site directed mutagenesis (Agilent QuikChange Lightning Site Directed Mutagenesis kit) to contain BpU10I restriction sites flanking the multiple cloning site. Plasmids were then grown in XL-10 GOLD *E.coli* and isolated using Nucleobond Midiprep kit (Macherey-Nagel), snap frozen and stored in aliquots at -80°C for future use. 80U of *M.SssI* CpG methyltransferase (Thermo Fisher) was used to methylate 12μg of plasmid, with 400μM SAM in a 100μl reaction alongside mock treated plasmids which were incubated in the same conditions without the *M.SssI* enzyme. To remove DNA with non-canonical bases (such as uracil or 8-oxoguanine), abasic sites and single strand breaks, 8μg of plasmid was incubated with 10U of UDG (NEB) and 16U FPG (NEB) in a 50μl solution with 0.5x UDG buffer and 0.5X NEB1 buffer for 30min, after which reaction volumes were increased to 100μl with 10μl 10x NEB4 (to a final concentration of 1x), 40U of T5 exonuclease (NEB) and water followed by a further incubation at 37°C for 30 minutes and purification on Serapure beads. 1 μg of plasmid was then nicked twice on the same strand by incubation with 1U of *Nt.BpU10I* (Thermo Fisher) in a 50μl reaction containing 1X buffer R at 37°C for one hour. Plasmid was then gapped by addition of 50μl 1X buffer R containing DNA oligonucleotides complimentary to the nicked strand at a 50-molar excess to the plasmid before incubating at 95°C - 1s, 60°C - 30s, 37°C - 1min (in PCR cycler). Non-plasmid DNA was removed using Serapure size selection. This step was repeated and full gapping confirmed by testing resistance to restriction digestion by *HindIII* (NEB) and *SacI* (NEB) (Extended Data Fig 1a). To exclude any non-gapped plasmid from participating in the

subsequent steps, samples were digested with 1U *BseRI* (NEB) per 1μg of plasmid (cuts both the p53 and DNMT ROI).

Supplementary Note 12: Introducing P286R mutation in mESCs

E14 mESC cells were grown on plates coated with 0.1% gelatin in water (Stemcell Technoligies) using DMEM, high glucose-10 media (Thermo Fisher Scientific) complemented with 15% (v/v) fetal bovine serum (Gibco,Thermo Fisher Scientific, Ref 10500-064, lot# 2534384H), 200 mM l-glutamine (Thermo Fisher Scientific), 1%(v/v) nonessential amino acids (Thermo Fisher Scientific), 1%(v/v) penicillin-streptomycin (Thermo Fisher Scientific), 50mM beta-mercaptoethanol (Thermo Fisher Scientific), 100 μg/ml leukemia inhibitory factor (LIF, produced in-house following a protocol by Tomala et al.[20]).

The POLE P286R was engineered in mESCs using CRISPR-Cas9 assisted homologous recombination. CRISPR guide RNA (Supplementary Table 8) was designed and cloned into the pX330 expression plasmid (Addgene #42230; containing the CRISPR-Cas9 system, eGFP and a G418-resistance marker) as previously described (Van Gool et al., 2018). Template for homologous recombination was single-stranded oligodeoxynucleotide (ssODNs) (Integrated DNA Technologies) designed to include the P286R mutation, a mutation in the protospacer adjacent motif (PAM) and a silent mutation to introduce *BbsI* restriction site (ssODNs in Supplementary Table 9).

60,000-70,000 cells were transfected with 1.5μg of pX330 and 3.5μg of ssODN using Lipofectamine 3000 (Thermo Fisher Scientific; L3000001). G418 (300μg/ml for 4 days; 4727878001, Merck Life Science UK) was added to the media for selection of transfected cells. The antibiotic-resistant cells were seeded at different concentrations into 100mm petri dishes and individual colonies were picked after 7-10 days into 96-well plate. To identify targeted clones, DNA was isolated and regions were amplified by PCR, followed by Sanger sequencing (Eurofins Genomics) (oligonucleotides sequences in Supplementary Table 10).

Expression levels of mutant DNA polymerase was measured by Western Blotting. Cells were lysed in RIPA lysis buffer (Thermo Fisher Scientific; 89901) supplemented with protease and phosphatase inhibitors (Roche) and protein concentration was determined using a BCA protein assay kit (Pierce). Lysates were denatured for 5 min at 95 °C in Laemmli buffer containing 10% beta-mercaptoethanol and subsequently electrophoresed on 4–12% precast polyacrylamide gels (NuPAGE™ Bis-Tris Mini Protein Gels; Invitrogen; NP0321BOX) under denaturing conditions. Proteins were wet-transferred onto PVDF using the Mini Trans-Blot Electrophoretic Transfer Cell System (Bio-Rad) at 100 V (400 mA) for 60 min at 4 °C. Membranes were blocked in 5% milk TBS-T for 1 hour at room temperature and subsequently incubated with anti-POLE (1:1000) (Stratech; GTX132100-GTX) and anti- β-actin (1:5000) (Cell Signaling Technology; 3700) overnight at 4 °C on a roller. After washing, membranes were probed

with either goat anti-mouse IgG (H + L)-HRP (Bio-Rad; 1706516) or goat anti-rabbit IgG (H + L)-HRP (Bio-Rad; 1706515) secondary antibodies diluted 1:2,500 for 45 minutes at room temperature. Membranes were developed with Pierce ECL (Thermo Fisher, 32106) and scanned using the ChemiDoc Imaging System (Bio-Rad).

Supplementary Note 13: PER-EXTRACT-seq

Preparation of nuclear extract and template filling was performed as described previously[21]. Briefly, 1-5x10^7 exponentially growing cells were detached by trypsinisation, pelleted by centrifugation at 400g for 3 min, washed in PBS and resuspended in 4ml of ice-cold Hypo/sucrose buffer (20mM HEPES-KOH pH 7.5, 5mM KCl, 1.5mM $MgCl_2$, 0.5mM DTT, 0.25M sucrose). Cells were the pelleted as above and buffer removed before resuspension in 4ml ice cold Hypo buffer (20mM HEPES-KOH pH 7.5, 5mM KCl, 1.5mM $MgCl_2$, 0.5mM DTT), centrifugation and final resuspension in 1ml of ice-cold Hypo buffer. Cells were allowed to swell on ice for 30min prior to disruption by ten strokes in an ice-cold dounce homogeniser (tight pestle) followed by another 30min incubation on ice. The suspension was clarified by centrifugation at 21 000g for 40min at 0°C after which the supernatant was split into 50µl aliquots, snap frozen and stored at -80°C.

Filling reactions were performed by gentle defrosting of nuclear extract and centrifugation at 21 000g at 0°C for 30min, followed by transfer of supernatant to an ice cold 1.5ml tube containing 50µl 2x filling buffer (60mM HEPES-KOH pH7.5, 14mM $MgCl_2$, 1mM DTT, 0.2µM dNTPs, 8mM ATP, 80mM phosphocreatine disodium hydrate (Merck Life Science UK Limited), 1mg of creatine phosphokinase type I from rabbit (Merck Life Science UK Limited) and 100ng of plasmid and reactions mixed by pipetting. Samples were then incubated at 37°C shaking at 220rpm for 5min before addition of 3µl 0.5M EDTA and 1µl proteinase K (20mg/ml). Reactions were incubated at 37°C for 20 min and DNA was then purified on Serapure beads as described previously. The PER-EXTRACT-seq samples are listed in Supplementary Table 11.

References used in the supplementary materials

1. Jee, Rasouly, Shamovsky, Akivis, R. Steinman, Mishra & Nudler. Rates and mechanisms of bacterial mutagenesis from maximum-depth sequencing. *Nature* **534**, 693–696 (2016).

2. Minoche, Dohm & Himmelbauer. Evaluation of genomic high-throughput sequencing data generated on Illumina HiSeq and Genome Analyzer systems. *Genome Biol* **12**, 1–15 (2011).

3. Ehrlich, Norris, Wang, Kuo & Gehrke. DNA cytosine methylation and heat-induced deamination. *Biosci Rep* **6**, 387–93 (1986).

4. Crevel, Kearsey & Cotterill. A simple bypass assay for DNA polymerases shows that cancer-associated hypermutating variants exhibit differences in vitro. *FEBS Journal* **290**, 5744–5758 (2023).

5. Haradhvala *et al.* Distinct mutational signatures characterize concurrent loss of polymerase proofreading and mismatch repair. *Nat Commun* **9**, 1746 (2018).

6. Campbell *et al.* Comprehensive Analysis of Hypermutation in Human Cancer. *Cell* **171**, 1042-1056.e10 (2017).

7. Church *et al.* DNA polymerase ε and δ exonuclease domain mutations in endometrial cancer. *Hum Mol Genet* **22**, 2820–2828 (2013).

8. Shinbrot *et al.* Exonuclease mutations in DNA Polymerase epsilon reveal replication strand specific mutation patterns and human origins of replication. *Genome Res* 1740–1750 (2014).

9. Campbell *et al.* Pan-cancer analysis of whole genomes. *Nature* **578**, 82–93 (2020).

10. Ahn *et al.* The somatic POLE P286R mutation defines a unique subclass of colorectal cancer featuring hypermutation, representing a potential genomic biomarker for immunotherapy. *Oncotarget* **7**, 68638–68649 (2016).

11. Kane & Shcherbakova. A common cancer-associated DNA polymerase ε mutation causes an exceptionally strong mutator phenotype, indicating fidelity defects distinct from loss of proofreading. *Cancer Res* **74**, 1895–1901 (2014).

12. Xing, Kane, Bulock, Moore, Sharma, Chabes & Shcherbakova. A recurrent cancer-associated substitution in DNA polymerase ε produces a hyperactive enzyme. *Nat Commun* **10**, 374 (2019).

13. Parkash, Kulkarni, ter Beek, Shcherbakova, Kamerlin & Johansson. Structural consequence of the most frequently recurring cancer-associated substitution in DNA polymerase ε. *Nat Commun* **10**, 373 (2019).

14. Soriano *et al.* Expression of the cancer-associated DNA polymerase ε P286R in fission yeast leads to translesion synthesis polymerase dependent hypermutation and defective DNA replication. *PLoS Genet* **17**, (2021).

15. Park & Pursell. POLE proofreading defects: Contributions to mutagenesis and cancer. *DNA Repair (Amst)* vol. 76 50–59 (2019).

16. Galati *et al.* Cancers from Novel Pole-Mutant Mouse Models Provide Insights into Polymerase-Mediated Hypermutagenesis and Immune Checkpoint Blockade. *Cancer Res* **80**, 5606–5618 (2020).

17. Li *et al.* Polymerase-mediated ultramutagenesis in mice produces diverse cancers with high mutational load. *J Clin Invest* **128**, 4179–4191 (2018).

18. Alexandrov, Jones, Wedge, Sale & Peter. Clock-like mutational processes in human somatic cells. *Nature* **47**, 1402–1407 (2015).

19. Alexandrov *et al.* The repertoire of mutational signatures in human cancer. *Nature* **578**, 94–101 (2020).

20. Tomala *et al.* Preparation of bioactive soluble human leukemia inhibitory factor from recombinant Escherichia coli using thioredoxin as fusion partner. *Protein Expr Purif* **73**, 51–57 (2010).

21. Oda, Humbert, Fiumicino, Bignami & Karran. Efficient repair of A/C mismatches in mouse cells deficient in long-patch mismatch repair. *EMBO Journal* **19**, 1711–1718 (2000).
