# Supplementary material for: Human DNA polymerase ε is a source of C>T mutations at CpG dinucleotides
Source: Nat Genet. 2024 Oct 10;56(11):2506–16. doi: 10.1038/s41588-024-01945-x (PMC11549043; doi:10.1038/s41588-024-01945-x)
Supplement: Supplementary file 2 — Reporting Summary [file 41588_2024_1945_MOESM2_ESM.pdf]

nature portfolio

Corresponding author(s): Marketa Tomkova, Michael McClellan, Benjamin Schuster-Böckler and Skirmantas Kriaucionis

Last updated by author(s): Aug 13, 2024

Reporting Summary

Nature Portfolio wishes to improve the reproducibility of the work that we publish. This form provides structure for consistency and transparency in reporting. For further information on Nature Portfolio policies, see our Editorial Policies and the Editorial Policy Checklist.

Statistics

For all statistical analyses, confirm that the following items are present in the figure legend, table legend, main text, or Methods section.

n/a	Confirmed	
☐	☒	The exact sample size (*n*) for each experimental group/condition, given as a discrete number and unit of measurement
☐	☒	A statement on whether measurements were taken from distinct samples or whether the same sample was measured repeatedly
☐	☒	The statistical test(s) used AND whether they are one- or two-sided<br>*Only common tests should be described solely by name; describe more complex techniques in the Methods section.*
☐	☒	A description of all covariates tested
☐	☒	A description of any assumptions or corrections, such as tests of normality and adjustment for multiple comparisons
☐	☒	A full description of the statistical parameters including central tendency (e.g. means) or other basic estimates (e.g. regression coefficient) AND variation (e.g. standard deviation) or associated estimates of uncertainty (e.g. confidence intervals)
☐	☒	For null hypothesis testing, the test statistic (e.g. *F*, *t*, *r*) with confidence intervals, effect sizes, degrees of freedom and *P* value noted<br>*Give P values as exact values whenever suitable.*
☒	☐	For Bayesian analysis, information on the choice of priors and Markov chain Monte Carlo settings
☒	☐	For hierarchical and complex designs, identification of the appropriate level for tests and full reporting of outcomes
☒	☐	Estimates of effect sizes (e.g. Cohen's *d*, Pearson's *r*), indicating how they were calculated

Our web collection on statistics for biologists contains articles on many of the points above.

Software and code

Policy information about availability of computer code

Data collection

No software was used for data collection (the data sets were downloaded using standard tools, such as wget).

Data analysis

Most of the data analysis was carried out using MATLAB (2022a).

The following MATLAB toolboxes have been used:
- Statistics and Machine Learning Toolbox (version 12.3)

The following MATLAB library has been used:
- lbmap: Robert Bemis (2022). Light Bartlein Color Maps (https://www.mathworks.com/matlabcentral/fileexchange/17555-light-bartlein-color-maps), MATLAB Central File Exchange. Retrieved June 23, 2022.

The other software used:
- bedtools (version 2.27.0)
- fastqc (version 0.11.8)
- bowtie2 (version 2.3.5.1)
- samtools (version 1.9)
- GNU Wget 1.14
- picard (version 2.21.6)
- trimgalore (version 0.4.5)
- bismark (version 0.19.0)

The code developed in this study is available at https://bitbucket.org/licroxford/per-seq (PER-seq pipeline) and https://bitbucket.org/licroxford/cpg_mutagenesis (analysis and comparison of PER-seq and cancer data, all code to reconstruct the figures and tables in the study).

For manuscripts utilizing custom algorithms or software that are central to the research but not yet described in published literature, software must be made available to editors and reviewers. We strongly encourage code deposition in a community repository (e.g. GitHub). See the Nature Portfolio guidelines for submitting code & software for further information.

Data

Policy information about availability of data

All manuscripts must include a data availability statement. This statement should provide the following information, where applicable:

- Accession codes, unique identifiers, or web links for publicly available datasets
- A description of any restrictions on data availability
- For clinical datasets or third party data, please ensure that the statement adheres to our policy

PER-seq sequencing data have been deposited in the Sequence Read Archive (SRA) under accession number SRP439101 and the processed files are available together with the code in the Bitbucket repositories. The used publicly available cancer samples are listed in Extended Data Table 5.

Research involving human participants, their data, or biological material

Policy information about studies with human participants or human data. See also policy information about sex, gender (identity/presentation), and sexual orientation and race, ethnicity and racism.

Reporting on sex and gender	NA
Reporting on race, ethnicity, or other socially relevant groupings	NA
Population characteristics	NA
Recruitment	NA
Ethics oversight	NA

Note that full information on the approval of the study protocol must also be provided in the manuscript.

Field-specific reporting

Please select the one below that is the best fit for your research. If you are not sure, read the appropriate sections before making your selection.

☒ Life sciences ☐ Behavioural & social sciences ☐ Ecological, evolutionary & environmental sciences

For a reference copy of the document with all sections, see nature.com/documents/nr-reporting-summary-flat.pdf

Life sciences study design

All studies must disclose on these points even when the disclosure is negative.

Sample size	Sample sizes indicated in individual figures, which represent typical values used in the field for this type of experiments. No sample size calculation was performed, however sample size is sufficient based on significance of relevant statistical tests applied.
Data exclusions	None of the experiments passing the technical experiment success criteria were excluded from the analyses
Replication	Experiments were replicated as indicated in the manuscript, appropriate statistical tests were used, and distributions and P values indicated.
Randomization	Randomization experiment design is not suitable for the presented experiments, because biochemical experiments do not employ sampling from the population.
Blinding	Blinding experiment design is not suitable for the presented experiments, because this study does not evaluate the effects of an exposure.

Reporting for specific materials, systems and methods

We require information from authors about some types of materials, experimental systems and methods used in many studies. Here, indicate whether each material, system or method listed is relevant to your study. If you are not sure if a list item applies to your research, read the appropriate section before selecting a response.

Materials & experimental systems

n/a	Involved in the study
☐	☒ Antibodies
☐	☒ Eukaryotic cell lines
☒	☐ Palaeontology and archaeology
☒	☐ Animals and other organisms
☒	☐ Clinical data
☒	☐ Dual use research of concern
☒	☐ Plants

Methods

n/a	Involved in the study
☒	☐ ChIP-seq
☒	☐ Flow cytometry
☒	☐ MRI-based neuroimaging

Antibodies

Antibodies used: POLE (Stratech; GTX132100-GTX); β-actin (Cell Signaling Technology; 3700); anti-mouse IgG (H + L)-HRP (Bio-Rad; 1706516); goat anti-rabbit IgG (H + L)-HRP (Bio-Rad; 1706515)

Validation: Multiple publications: Nature (PMID: 37968395), Cell Reports (PMID: 35649380), Cell (PMID: 35512704), J Med Gen (PMID: 35534205)

Eukaryotic cell lines

Policy information about cell lines and Sex and Gender in Research

Cell line source(s): E14 mESCs gift from Adrian Bird group, HCC2998 gift from Xin Lu group

Authentication: Non authenticated, WGS data provided for mESCs and could be used for verification of validity.

Mycoplasma contamination: Cells were regularly (monthly) tested for mycoplasma and found negative

Commonly misidentified lines (See ICLAC register): None

Plants

Seed stocks: *Report on the source of all seed stocks or other plant material used. If applicable, state the seed stock centre and catalogue number. If plant specimens were collected from the field, describe the collection location, date and sampling procedures.*

Novel plant genotypes: *Describe the methods by which all novel plant genotypes were produced. This includes those generated by transgenic approaches, gene editing, chemical/radiation-based mutagenesis and hybridization. For transgenic lines, describe the transformation method, the number of independent lines analyzed and the generation upon which experiments were performed. For gene-edited lines, describe the editor used, the endogenous sequence targeted for editing, the targeting guide RNA sequence (if applicable) and how the editor was applied.*

Authentication: *Describe any authentication procedures for each seed stock used or novel genotype generated. Describe any experiments used to assess the effect of a mutation and, where applicable, how potential secondary effects (e.g. second site T-DNA insertions, mosiacism, off-target gene editing) were examined.*
