# Supplementary figures and images for: Human DNA polymerase ε is a source of C>T mutations at CpG dinucleotides
Source: Nat Genet. 2024 Oct 10;56(11):2506–16. doi: 10.1038/s41588-024-01945-x (PMC11549043; doi:10.1038/s41588-024-01945-x)

Raw Fig 1a

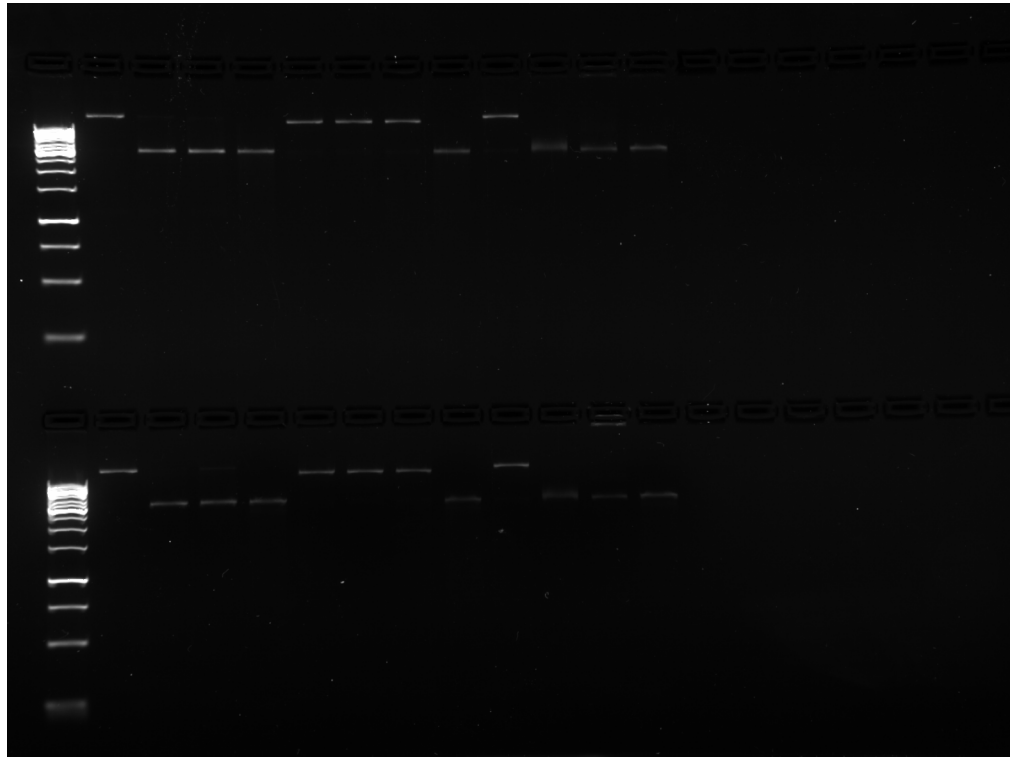

Raw Fig 1c

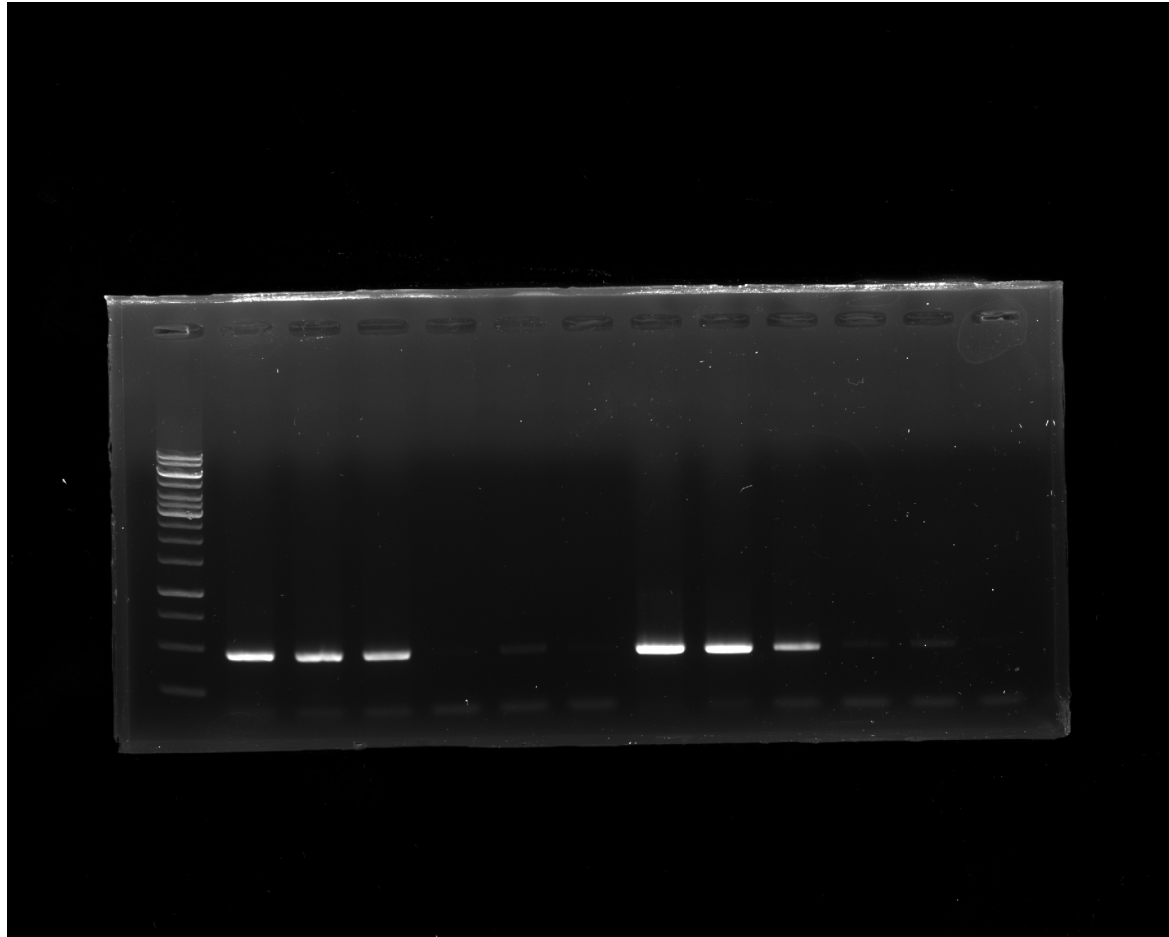

Raw Fig 1e

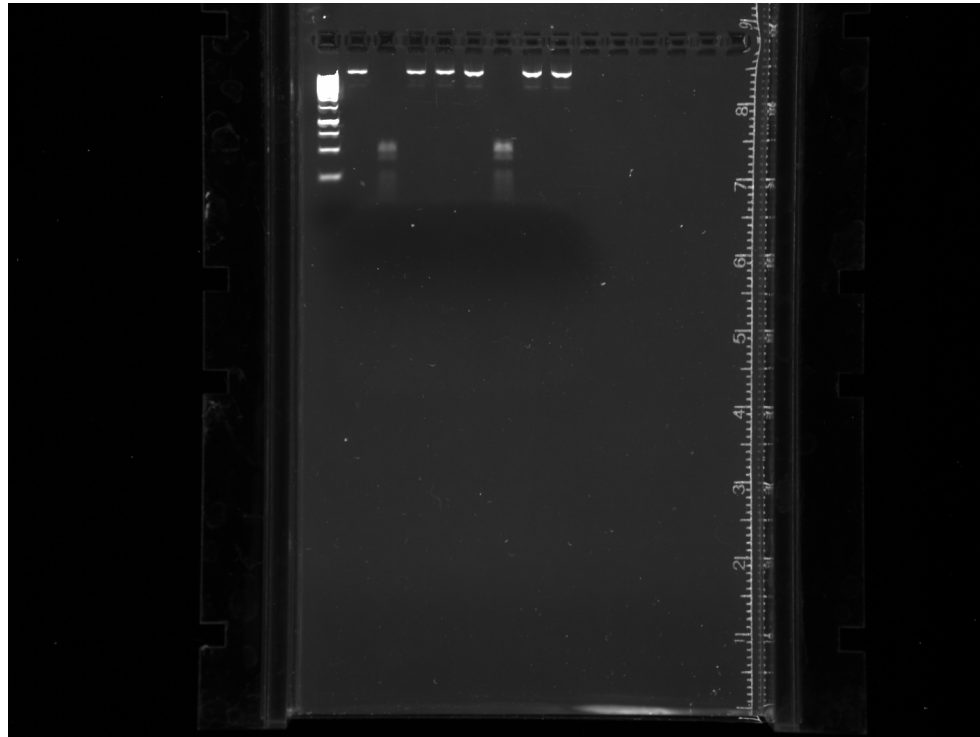

Supplement: Supplementary file 5 — Unprocessed western blots and gels. [file 41588_2024_1945_MOESM5_ESM.pdf]

Raw Fig 5c

POLE Ladder

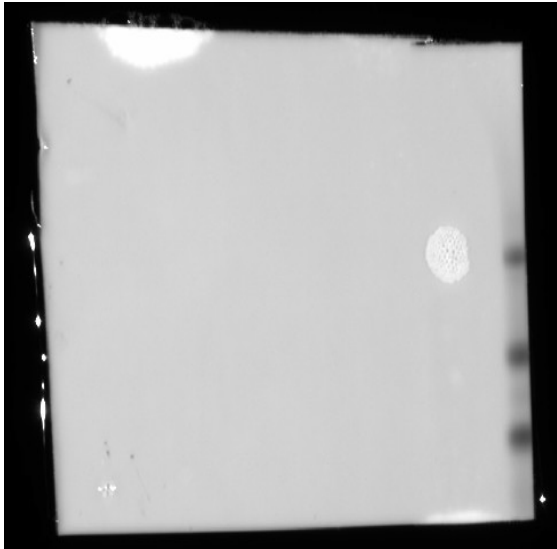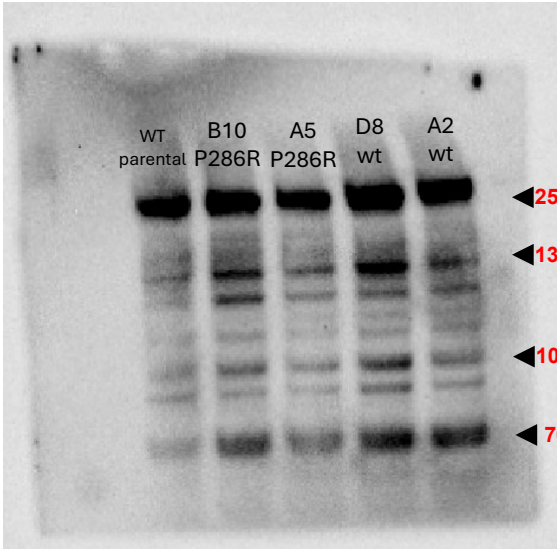

POLE (260 kDa)

◀ 250 kDa

◀ 130 kDa

◀ 100 kDa

◀ 70 kDa

β-actin Ladder

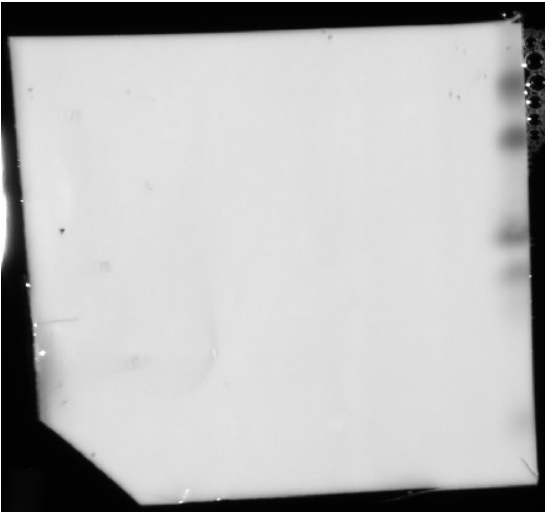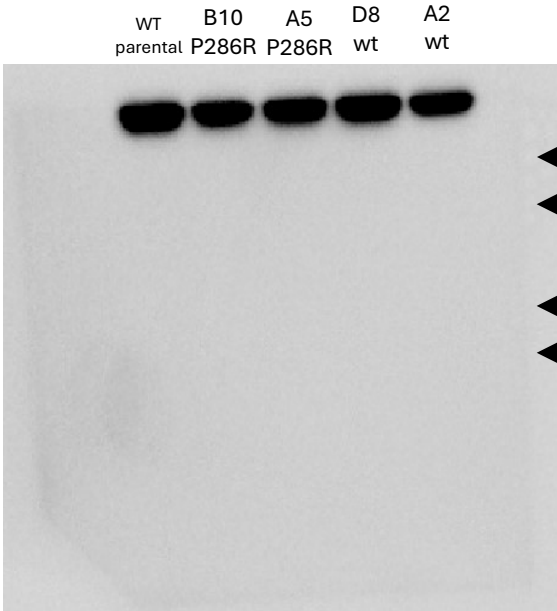

β-actin (42 kDa)

◀ 35 kDa

◀ 25 kDa

◀ 15 kDa

◀ 10 kDa

Raw Fig 5d

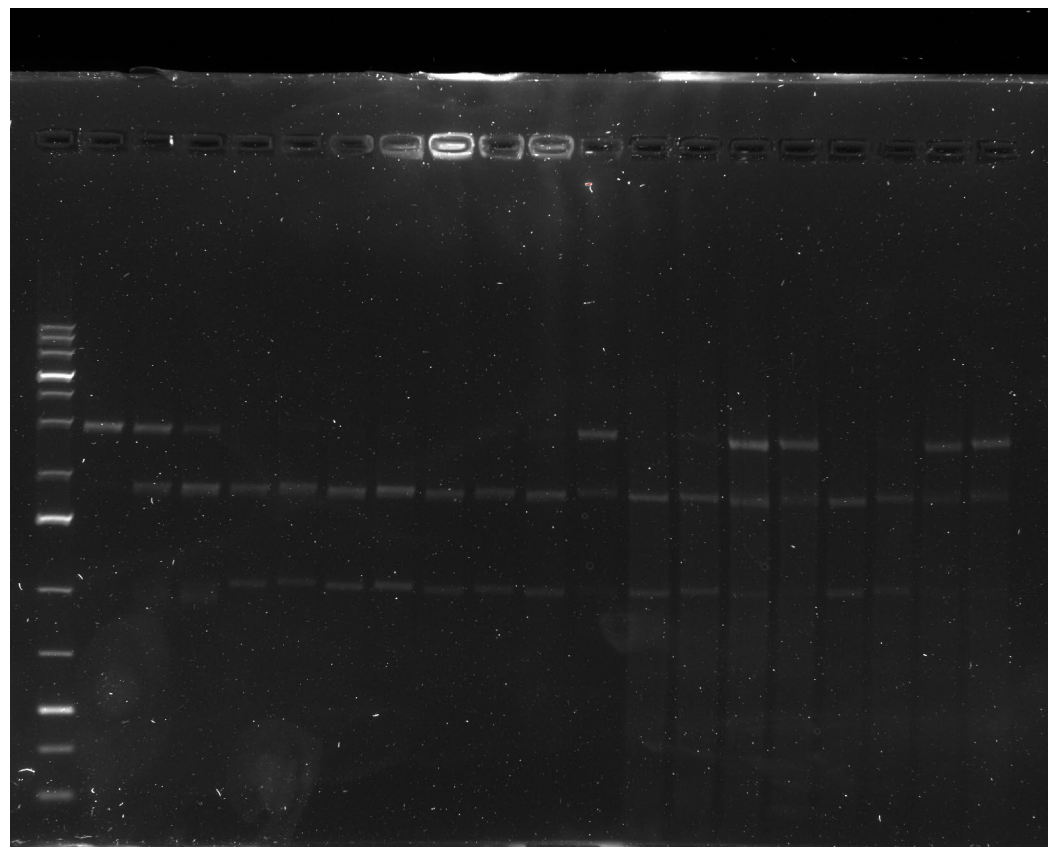

Supplement: Supplementary file 6 — Unprocessed gels. [file 41588_2024_1945_MOESM6_ESM.pdf]
